# Coffee and tea consumption and risk of pre- and postmenopausal breast cancer in the European Prospective Investigation into Cancer and Nutrition (EPIC) cohort study

**DOI:** 10.1186/s13058-015-0521-3

**Published:** 2015-01-31

**Authors:** Nirmala Bhoo-Pathy, Petra HM Peeters, Cuno SPM Uiterwaal, H Bas Bueno-de-Mesquita, Awang M Bulgiba, Bodil Hammer Bech, Kim Overvad, Anne Tjønneland, Anja Olsen, Françoise Clavel-Chapelon, Guy Fagherazzi, Florence Perquier, Birgit Teucher, Rudolf Kaaks, Madlen Schütze, Heiner Boeing, Pagona Lagiou, Philippos Orfanos, Antonia Trichopoulou, Claudia Agnoli, Amalia Mattiello, Domenico Palli, Rosario Tumino, Carlotta Sacerdote, Franzel JB van Duijnhoven, Tonje Braaten, Eiliv Lund, Guri Skeie, María-Luisa Redondo, Genevieve Buckland, Maria José Sánchez Pérez, Maria-Dolores Chirlaque, Eva Ardanaz, Pilar Amiano, Elisabet Wirfält, Peter Wallström, Ingegerd Johansson, Lena Maria Nilsson, Kay-Tee Khaw, Nick Wareham, Naomi E Allen, Timothy J Key, Sabina Rinaldi, Isabelle Romieu, Valentina Gallo, Elio Riboli, Carla H van Gils

**Affiliations:** Julius Center for Health Sciences and Primary Care, University Medical Center, P.O. Box 85500, 3508 GA Utrecht, The Netherlands; Department of Social and Preventive Medicine, Faculty of Medicine, Julius Centre University of Malaya, University of Malaya, Lembah Pantai, Kuala Lumpur, Malaysia; National Clinical Research Centre, Kuala Lumpur Hospital, Ministry of Health, Kuala Lumpur, Malaysia; School of Public Health, Imperial College London, London, UK; National Institute of Public Health and the Environment (RIVM), Bilthoven, The Netherlands; Department of Gastroenterology and Hepatology, University Medical Centre, Utrecht, The Netherlands; Department of Public Health, Section for Epidemiology, Aarhus University, Aarhus, Denmark; Danish Cancer Society, Institute of Cancer Epidemiology, Copenhagen, Denmark; Inserm, Centre for Research in Epidemiology and Population Health (CESP), U1018, “Nutrition, Hormones, and Women’s Health” Team, Institut Gustave Roussy, F-94805 Villejuif, France; Université Paris Sud 11, UMRS 1018, F-94807 Villejuif, France; Department of Cancer Epidemiology, German Cancer Research Center, Heidelberg, Germany; Department of Epidemiology, German Institute of Human Nutrition, Potsdam-Rehbruecke, Germany; Department of Hygiene, Epidemiology and Medical Statistics, WHO Collaborating Center for Food and Nutrition Policies, University of Athens Medical School, 75 M. Asias Avenue, Goudi, GR-115 27 Athens, Greece; Hellenic Health Foundation, 10-12 Tetrapoleos Street, GR-115 27 Athens, Greece; Nutritional Epidemiology Unit, Fondazione IRCCS Istituto Nazionale Tumori, Via Venezian, 1, 20133 Milan, Italy; Dipartimento di Medicina Clinica e Chirurgia, University of Naples Federico II, Via Pansini, 5 80131 Naples, Italy; Molecular and Nutritional Epidemiology Unit, Cancer Research and Prevention Institute – ISPO, Florence, Italy; Cancer Registry and Histopathology Unit, “Civile - M.P.Arezzo” Hospital, ASP 7, Ragusa, Italy; HuGeF Foundation and CPO-Piemonte, Torino, Italy; Division of Human Nutrition, Wageningen University, Wageningen, The Netherlands; Department of Community Medicine, Faculty of Health Sciences, University of Tromsø, The Arctic University of Norway, Tromsø, Norway; Public Health and Participation Directorate, Health and Health Care Services Council, Asturias, Spain; Unit of Nutrition, Environment and Cancer, Cancer Epidemiology Research Programme, Catalan Institute of Oncology (ICO-IDIBELL), Barcelona, Spain; Escuela Andaluza de Salud Pública, Instituto de Investigación Biosanitaria de Granada (Granada.ibs), Granada, Spain; Consortium for Biomedical Research in Epidemiology and Public Health (CIBER Epidemiología y Salud Pública-CIBERESP), Madrid, Spain; Department of Epidemiology, Murcia Health Council, Murcia, Spain; Navarre Public Health Institute, Pamplona, Spain; Public Health Division of Gipuzkoa, Instituto Investigación Sanitaria, San Sebastian, Spain; Department of Clinical Sciences in Malmö/Nutrition Epidemiology, Lund University, Malmö, Sweden; Department of Odontology, Umeå University, Umeå, Sweden; Department of Public Health and Clinical Medicine, Nutritional Research, Umeå University, Umea, Sweden; University of Cambridge School of Clinical Medicine, Cambridge, UK; Medical Research Council, Epidemiology Unit, Cambridge, UK; Cancer Epidemiology Unit, University of Oxford, Richard Doll Building, Roosevelt Drive, Oxford, OX3 7LF UK; International Agency for Research on Cancer, Lyon, France; Centre for Primary Care and Public Health, Barts and The London School of Medicine, Queen Mary University of London, London, UK

## Abstract

**Introduction:**

Specific coffee subtypes and tea may impact risk of pre- and post-menopausal breast cancer differently. We investigated the association between coffee (total, caffeinated, decaffeinated) and tea intake and risk of breast cancer.

**Methods:**

A total of 335,060 women participating in the European Prospective Investigation into Nutrition and Cancer (EPIC) Study, completed a dietary questionnaire from 1992 to 2000, and were followed-up until 2010 for incidence of breast cancer. Hazard ratios (HR) of breast cancer by country-specific, as well as cohort-wide categories of beverage intake were estimated.

**Results:**

During an average follow-up of 11 years, 1064 premenopausal, and 9134 postmenopausal breast cancers were diagnosed. Caffeinated coffee intake was associated with lower risk of postmenopausal breast cancer: adjusted HR = 0.90, 95% confidence interval (CI): 0.82 to 0.98, for high versus low consumption; *P*_trend_ = 0.029. While there was no significant effect modification by hormone receptor status (*P* = 0.711), linear trend for lower risk of breast cancer with increasing caffeinated coffee intake was clearest for estrogen and progesterone receptor negative (ER-PR-), postmenopausal breast cancer (*P* = 0.008). For every 100 ml increase in caffeinated coffee intake, the risk of ER-PR- breast cancer was lower by 4% (adjusted HR: 0.96, 95% CI: 0.93 to 1.00). Non-consumers of decaffeinated coffee had lower risk of postmenopausal breast cancer (adjusted HR = 0.89; 95% CI: 0.80 to 0.99) compared to low consumers, without evidence of dose–response relationship (*P*_trend_ = 0.128). Exclusive decaffeinated coffee consumption was not related to postmenopausal breast cancer risk, compared to any decaffeinated-low caffeinated intake (adjusted HR = 0.97; 95% CI: 0.82 to 1.14), or to no intake of any coffee (HR: 0.96; 95%: 0.82 to 1.14). Caffeinated and decaffeinated coffee were not associated with premenopausal breast cancer. Tea intake was neither associated with pre- nor post-menopausal breast cancer.

**Conclusions:**

Higher caffeinated coffee intake may be associated with lower risk of postmenopausal breast cancer. Decaffeinated coffee intake does not seem to be associated with breast cancer.

**Electronic supplementary material:**

The online version of this article (doi:10.1186/s13058-015-0521-3) contains supplementary material, which is available to authorized users.

## Introduction

Coffee and tea are the most popular beverages consumed worldwide, rendering them as relevant dietary exposures [1]. Coffee and tea consumption may protect against breast cancer through anticarcinogenic properties of their biochemical compounds such as caffeine, polyphenols and diterpenes [2-4] or through favorably altering the levels of hormones implicated in breast cancer [5-9]. While polyphenols, including flavonoids, may mimic estradiol structure and, hence, antagonize estrogen action, paradoxically, they may also bind weakly to estrogen receptors and promote estrogen-dependent transcription [10].

A major systematic review by the World Cancer Research Fund and American Institute for Cancer Research had concluded that for the association between coffee and tea intake and pre- and postmenopausal breast cancer, evidence did not allow for definite conclusions [11]. A minority of studies that distinguished between types of coffee consumed showed contradictory results for decaffeinated coffee [12]. Nevertheless, it is conceivable that different types of coffee are associated with opposing effects on cancer risk owing to differences in their constituents. For instance, decaffeinated coffee may contain very low levels of caffeine (up to 0.1%) [13]. Therefore, it is pertinent to further explore the effects arising from differing caffeine levels in caffeinated and decaffeinated coffee.

Premenopausal and postmenopausal breast cancers have been argued for some time to be diseases with somewhat different etiologies [14,15], and it is conceivable that dietary factors may impact the risk of pre- and postmenopausal breast cancer differently [16]. It has also been recently hypothesized that breast cancer comprises two fundamental etiological components, which are to a certain extent defined by estrogen receptor expression by age at diagnosis. Therefore, it has been proposed that in large-scale population based studies, etiological analyses for breast cancer should be stratified according to molecular subtypes [17]. To date, relatively few studies have differentiated between pre- and postmenopausal breast cancers [12,18,19] or investigated the association between coffee and tea intake with breast cancer based on estrogen receptor (ER) and progesterone receptor (PR) status [12,18,19]. The results have been overall inconsistent and may be attributed to the fact that most studies were hampered by limited numbers of cases [12,18,20].

We determined the association between coffee (total, decaffeinated and caffeinated) and tea consumption with risk of pre- and postmenopausal breast cancer within the European Prospective Investigation into Cancer and Nutrition (EPIC) cohort [21]. Distinction was also made between breast cancers by hormone receptor status.

## Methods

The EPIC study is an on-going multi-center prospective cohort study aimed at investigating the association between diet, lifestyle, genetic and environmental factors and the development of cancer and other chronic diseases. It consists of 521,448 men and women, followed-up for cancer incidence and cause-specific mortality for several decades. There are 23 EPIC centers in 10 European countries, that is, Denmark, France, Germany, Greece, Italy, Netherlands, Norway, Spain, Sweden, and United Kingdom. Details have been described elsewhere [21]. At enrollment between 1992 and 2000, information on habitual diet in the preceding year was collected through a questionnaire in most countries. Lifestyle questionnaires were used for information on education, reproductive history, use of oral contraceptives and hormone therapy, family history, medical history, physical activity and history of consumption of alcohol and tobacco [21].

### Study participants

This study pertains to female participants of the EPIC cohort between 25- and 70-years old at recruitment. We excluded participants with prior history of cancer, incomplete dietary/non-dietary information, and poorly completed questionnaires based on their ratio of energy intake versus energy expenditure (bottom 1% or top 1% of the cohort), leaving 335,060 women.

All participants provided written informed consent. The study was approved by the International Agency for Research on Cancer (IARC)’s ethical review committee and by the respective local ethical committees.

### Exposure assessment

Diet was assessed using country-specific questionnaires [21], namely self-administered semi-quantitative food-frequency questionnaires (±260 food items), dietary history questionnaires (>600 food items) administered by interviewers, and semi-quantitative food-frequency questionnaires combined with a food record. Further details on questionnaires and their validation are described elsewhere [22].

As the exact structure of the questions varied by center and questionnaire, complete information on caffeinated and decaffeinated coffee intake was available only in Germany, Greece, Italy (except Ragusa and Naples), the Netherlands, Norway, Spain, Sweden (except Umea), and the United Kingdom. Analyses of caffeinated and decaffeinated coffee consumption only included women with complete information on type of coffee intake, that is, those whose different types of coffee intakes added up to their total coffee intake. For caffeinated coffee consumption, 226,368 participants were included. Since none of the participants in Norway and Sweden consumed decaffeinated coffee, they were excluded from analysis for decaffeinated coffee consumption, leaving 176,373 participants. Information on tea intake was not available in Norway, leaving 299,890 participants.

As the cohort consists of multiple populations with a wide range of variation in terms of volume and concentration of coffee and tea intake, country specific quartiles for these beverages were estimated based on distribution of intake within each country, after excluding the non-consumers. This yielded the following intake categories for total coffee, caffeinated coffee and tea: none, low, moderately low, moderately high, and high. As decaffeinated coffee intake was less common, we used tertiles of intake for the consumers and intake categories were: none, low, medium, and high.

### Ascertainment of breast cancer cases

The outcome of interest was first incident of primary invasive breast cancer (coded using International Classification of Diseases for Oncology, Second Edition as C50.0-C50.9). As data on menopausal status at diagnosis was lacking, breast cancers occurring before the median menopausal age of 50 years were considered premenopausal, whereas those diagnosed at 50 years or older were considered postmenopausal. Information on hormone receptor status was provided by each center based on pathology reports. This information was routinely available for tumors diagnosed after 1997 to 2006, depending on the center.

### Follow-up

Follow-up was based on linkage with population cancer registries in Denmark, Italy, Netherlands, Norway, Spain, Sweden and the United Kingdom. In France, Germany and Greece, combined methods including health insurance records, cancer and pathology registries, and active follow-up were used. Censoring dates for most centers depended on the dates at which cancer registries were considered complete (varying from December 2004 in Spain to December 2008 in Italy). In Germany, Greece and France where active follow-up was undertaken, dates of censoring were up to March 2010, December 2009, and July 2005, respectively. Loss to follow-up was less than 4%.

### Statistical analysis

Multivariable Cox regression was used to examine the association between coffee or tea consumption and risk of breast cancer. Time at entry was age at recruitment, and exit time was age at diagnosis with breast cancer as first tumor, death, emigration, loss to follow-up, or end of follow-up. The non-zero slope of the scaled Schoenfeld residuals on the time function suggested that the proportional hazard assumption was met. All analyses were stratified by age at recruitment in one-year categories and by centers to control for differences in recruitment or follow-up procedures and questionnaire design. We studied consumption of beverages both as categorical and continuous (increment of 100 ml/day) variables. Non-consumers of coffee comprised a relatively small group (<10%) and seemed to have some unique health behaviors: they were less likely to have ever smoked, consume alcohol, or to have ever used oral contraceptives, and they were more likely to be physically inactive compared to the rest of the study population. We, therefore, used the low coffee consumers as the reference group in the categorical data analysis. To test for linear trends, the categories were entered as a continuous term (score variable: 0,1,2,3,4) in the Cox model. Since most coffee consumers tend to consume caffeinated as well as decaffeinated coffee, we additionally cross-classified coffee intakes in relation to breast cancer. This yielded eight categories, of which (any) decaffeinated coffee consumers with low caffeinated coffee intake comprised the largest group and was hence chosen as the reference for reasons of statistical robustness.

Two separate Cox models were fitted for pre- and postmenopausal breast cancers (Additional file 1). Both models were adjusted for age at menarche (categorical: <12, 12 to 4, >15 years), ever use of oral contraceptives (yes/no), age at first delivery (categorical: nulliparous, <20, 20 to 29, 30 to 39, ≥40 years), ever breastfeeding (yes/no), smoking status (categorical: never, past, current), educational level (categorical: none, primary school, technical/professional school, secondary school, university), physical activity level based on the Cambridge Physical Activity Index [23] (categorical: inactive, moderately inactive, moderately active, active), alcohol intake (continuous), height (continuous), weight (continuous), energy intake from fat source (continuous), energy intake from non-fat source (continuous), total saturated fat intake (continuous), and total fiber intake (continuous). The model for postmenopausal breast cancer was additionally adjusted for ever-use of postmenopausal hormones (yes/no). Importantly, coffee and tea intake were mutually adjusted for one another while models for caffeinated and decaffeinated coffee were also mutually adjusted.

As data on hormone receptor status was only available in breast cancer cases diagnosed after 1997 to 2006, depending on the center, it is mainly the postmenopausal cases that had receptor status available (77.2%) and only about half of the premenopausal cases (58.6%). Hormone-receptor defined analyses were, hence, only done among postmenopausal breast cancers and were not possible within premenopausal breast cancers.

As a form of sensitivity analysis, we also analyzed beverage intake using categories based on the overall cohort instead of country specific intake.

To improve comparability across centers, dietary intake was calibrated by a 24-hour dietary recall method common to all centers, in a random sub-sample of 8% of the cohort at baseline (Additional file 1) [24,25].

Heterogeneity of the association according to hormone receptor status was assessed using a data-augmentation method described by Lunn and McNeil [26]. Effect modification by body mass index [27] and country were assessed, and several sensitivity analyses were conducted (Additional file 1).

Two-tailed *P*-values <0.05 and 95% confidence intervals (CI) for hazard ratios (HRs) not including 1 were considered statistically significant. All analyses were performed using SAS version 9.1 (SAS Institute Inc, Cary, NC, USA).

## Results

A great majority of the study participants consumed coffee, with a median total coffee intake ranging from 93 ml/day in Italy to 900 ml/day in Denmark (not shown). Decaffeinated coffee was consumed by about 50% of the study population. Median decaffeinated coffee intake ranged from 2 ml/day in Spain and United Kingdom to 140 ml/day in France. Tea was consumed by approximately 66% of the total cohort resulting in a median intake ranging from close to 0 ml/day in Greece to up to 475 ml/day in United Kingdom.

The mean age at recruitment was 51 years with 43% of the participants being postmenopausal. Based on body mass index (BMI) classification by the World Health Organization, 58% of participants were of normal weight, 29% overweight and 13% obese.

Compared to the low coffee consumers, those with high coffee consumption were more likely to have ever smoked, used oral contraceptives, be physically active, and consumed more alcohol but less tea (Table 1). They were also more likely to comprise women attaining early menarche, and those with very young age at first childbirth (<20 years). In contrast, participants with high decaffeinated coffee intake were less likely to have used oral contraceptives, and more likely to be postmenopausal, and use hormone replacement therapy, compared to the low consumers. They also less frequently attained tertiary education, and were more likely to have breastfed their offspring. However, compared to non-consumers, consumers of (any) decaffeinated coffee were more likely to have attained tertiary education, be physically active, be non-smokers, and less likely to be very young at first childbirth (not shown). Compared to low tea intake, those with high tea intake were more likely to be older at recruitment, have higher education status, be more active physically, use more oral contraceptives, be older at first delivery, be postmenopausal, and use hormone replacement therapy.

**Table 1:** **Distribution of risk factors according to levels of consumption of coffee (total, caffeinated and decaffeinated) and tea**

	**Total**	**Coffee (total)** ^a^		**Coffee caffeinated** ^b^		**Coffee decaffeinated** ^c^		**Tea** ^d^	
		**Low intake** ^e^	**High intake** ^e^	**Low intake** ^e^	**High intake** ^e^	**Low intake** ^f^	**High intake** ^f^	**Low intake** ^e^	**High intake** ^e^
Age at recruitment (mean (years))	51	51	50	49	50	47	50	49	52
Familial breast cancer (%)^g^	8.3	8.6	7.9	9.5	9.0	10.6	9.9	9.6	9.3
Age at menarche (% <12 years)	15.0	13.6	17.1	13.3	15.6	16.5	17.5	15.8	14.7
Oral contraceptive use (% ever)	58.7	58.1	61.3	65.2	68.4	71.5	67.8	62.6	67.1
Nulliparity (%)	4.1	5.3	4.1	6.8	4.6	6.2	3.7	4.0	3.0
Age at first delivery (% < 20 years)^h^	14.8	13.4	18.1	13.2	18.0	10.1	11.1	15.2	12.3
Breastfed offsprings (% ever)	72.2	71.1	72.4	73.7	74.9	60.7	66.7	66.3	68.5
Postmenopausal (%)	43.4	43.1	38.5	38.6	38.5	34.9	42.0	40.4	45.7
Menopausal hormone use (% ever)	26.0	25.7	25.6	26.9	28.5	19.5	24.0	23.2	31.6
Education (% university)	23.6	25.1	22.7	28.6	23.4	37.6	29.1	33.2	37.7
Smokers (% ever)	42.0	35.6	54.6	40.5	57.6	40.3	43.6	43.5	42.1
Physically inactive^i^ (%)	24.3	25.6	22.8	19.3	19.1	19.4	18.9	21.2	16.4
BMI (mean (kg/m^2^))	25.0	24.8	25.2	24.2	24.9	24.2	24.9	24.8	24.1
Alcohol intake (median (g/day))	3.6	2.9	4.2	3.2	4.7	4.5	4.0	4.2	5.2
Energy intake (mean (kcal/day))	1931	1863	2008	1835	1968	1892	1919	1906	2003
Fat intake (mean (g/day))	25	24	26	23	24	22	22	25	23
Fruits intake (mean (g/day))	250	249	245	232	216	254	261	255	244
Vegetable intake (mean (g/day))	219	214	227	204	196	238	236	231	227
Tea^d^ intake (median (ml/day))	29	14	1	15	2	356	238	12	814
Coffee^j^ intake (median (ml/day))	290	70	750	2	0	190	81	376	150

^a^Includes all 335,060 participants. ^b^Includes 226,368 participants with complete data on type of coffee intake, that is, France (n = 48,101), Germany (n = 27,411), Greece (n = 3,125), Italy (n = 11,737), Netherlands (n = 26,866), Norway (n = 35,170), Spain (n = 6,589), Sweden (n = 14,825), and United Kingdom (n = 52,544). ^c^Includes 176,373 participants with complete data on type of coffee intake, that is, France (n = 48,101), Germany (n = 27,411), Greece (n = 3,125), Italy (n = 11,737), Netherlands (n = 26,866), Spain (n = 6,589), and United Kingdom (52,544). Participants from Norway and Sweden are all non-consumers of decaffeinated coffee and were excluded. ^d^Includes 299,890 participants. Participants from Norway were excluded as their information on tea intake is not available. ^e^Cut-off points are based on country specific quartiles of beverage intake after exclusion of non-consumers; low: quartile 1, high: quartile 4. ^f^Cut-off points are based on country specific tertiles of decaffeinated coffee intake after exclusion of non-consumers; low: tertile 1, high: tertile 3. ^g^In first degree relative (available for 43% of women). ^h^Only for parous women. ^i^Using Cambridge Physical Activity Index. ^j^For caffeinated coffee categories, median decaffeinated coffee intake is given and vice versa.

During an average 11 years of follow-up, 10,198 first incidences of primary invasive breast cancer were observed among 335,060 women. Of these, 1,064 were premenopausal breast cancers. Hormone receptor status was available in approximately 70% (7,053) of total breast cancer cases, out of which 50% were double hormone receptor positive tumors (ER+ PR+), followed by 33% of single hormone receptor positive tumors (ER+ or PR+), whereas 17% were double negative tumors (ER- PR-).

Tables 2, 3, 4, 5 and 6 show the numbers of participants, cases, and multivariable adjusted HRs for each category of coffee (total, caffeinated, decaffeinated intake) and tea intake. For analysis of beverages as continuous value (per 100 ml increment), the observed and calibrated HRs were identical. We only present the observed HR.

**Table 2:** **Total coffee consumption and risk of breast cancer** ^a^

**Daily coffee intake**	**Total**	**No intake**	**Low intake** ^b^	**Moderately low intake** ^b^	**Moderately high intake** ^b^	**High intake** ^b^	***P***_trend_^c^	**Per 100 mls**
Number of participants	335060	26734	87501	71684	79838	69303		
Number of breast cancers	10198	813	2542	2213	2518	2112		
**Premenopausal breast cancers**	1064	81	246	234	251	252		
Adjusted Hazard Ratio (95% CI)^d^		1.08 (0.83-1.40)	1.00	1.23 (1.02-1.48)	1.11 (0.93-1.34)	1.15 (0.96-1.39)	0.272	1.00 (0.98-1.03)
**Postmenopausal breast cancers**	9134	732	2296	1979	2267	1860		
Adjusted Hazard Ratio (95% CI)^e^		1.02 (0.94-1.12)	1.00	0.97 (0.91-1.03)	0.97 (0.92-1.03)	0.95 (0.89-1.01)	0.055	0.99 (0.98-0.99)
**ER+ and PR+ breast cancers**	3206	285	860	670	776	615		
Adjusted Hazard Ratio (95% CI)^f^		0.97 (0.84-1.11)	1.00	0.96 (0.86-1.06)	0.98 (0.88-1.08)	0.91 (0.81-1.01)	0.187	0.99 (0.97-1.00)
**ER- and PR- breast cancers**	1052	93	269	222	257	211		
Adjusted Hazard Ratio (95% CI)^g^		0.99 (0.78-1.26)	1.00	0.84 (0.70-1.01)	0.89 (0.74-1.06)	0.86 (0.71-1.05)	0.135	0.99 (0.97-1.01)
**Analysis by cohort-wide intake**								
Adjusted Hazard Ratio (95% CI)^h^		0.99 (0.76-1.29)	1.00	1.01 (0.84-1.20)	1.01 (0.83-1.83)	1.09 (0.88-1.35)	0.501	1.00 (0.98-1.03)
Adjusted Hazard Ratio (95% CI)^i^		1.03 (0.94-1.13)	1.00	0.99 (0.92-1.06)	0.98 (0.91-1.05)	0.95 (0.88-1.02)	0.067	0.99 (0.98-0.99)

^a^Includes all 335,060 participants. ^b^Cut-off points are based on country specific quartiles of total coffee intake after exclusion of non-coffee consumers. ^c^ *P* for trend is computed by entering the categories as a continuous term (score variable: 0,1,2,3,4) in the Cox model. ^d^Including only premenopausal breast cancers (that is, breast cancer diagnosed before the age of 50 years), and participants who were premenopausal at recruitment. Model is stratified by study center and age at recruitment, and adjusted for age at menarche, ever use of oral contraceptives, age at first delivery, breastfeeding, smoking, education, physical activity level, alcohol intake, height, weight, energy intake from fat sources, energy intake from non-fat sources, saturated fat intake, fruits and vegetable intake, and tea intake. ^e^Including only postmenopausal breast cancers (excluding participants with premenopausal breast cancers). Model is stratified by study center and age at recruitment, and adjusted for age at menarche, ever use of oral contraceptives, age at first delivery, breastfeeding, menopausal status at recruitment, ever use of postmenopausal hormones, smoking, education, physical activity level, alcohol intake, height, weight, energy intake from fat sources, energy intake from non-fat sources, saturated fat intake, fruits and vegetable intake, and tea intake. ^f^Hormone receptor status was only known in approximately 67% of patients with breast cancer. This analysis includes only estrogen receptor positive and progesterone receptor positive postmenopausal breast cancers, fully adjusted as in model 5. ^g^Hormone receptor status was only known in approximately 67% of patients with breast cancer. This analysis includes only estrogen receptor negative and progesterone receptor negative postmenopausal breast cancers, fully adjusted as in model 5. ^h^Including only premenopausal breast cancers. Using total coffee intake in cohort wide categories (no intake, quartile 1, quartile 2, quartile 3, quartile 4), and fully adjusted as in model 4. ^i^Including only postmenopausal breast cancers. Using total coffee intake in cohort wide categories (no intake, quartile 1, quartile 2, quartile 3, quartile 4), and fully adjusted as in model 5. CI, confidence interval; ER, estrogen receptor; PR, progesterone receptor.

**Table 3:** **Caffeinated coffee consumption and risk of breast cancer** ^a^

**Daily caffeinated coffee intake**	**Total**	**No intake of caffeinated coffee**	**Low intake** ^b^	**Moderately low intake** ^b^	**Moderately high intake** ^b^	**High intake** ^b^	***P***_trend_^c^	**Per 100 mls**
No. of participants	226 368	35590	60772	46429	43565	40012		
No. of breast cancers	6794	1068	1783	1360	1356	1227		
**Premenopausal breast cancers**	724	102	189	159	133	141		
Adjusted Hazard Ratio (95% CI)^d^		1.14 (0.86-1.53)	1.00	1.23 (0.97-1.55)	1.01 (0.79-1.28)	1.19 (0.93-1.53)	0.547	1.00 (0.97-1.03)
**Postmenopausal cancers**	6070	966	1594	1201	1223	1086		
Adjusted Hazard Ratio (95% CI)^e^		1.00 (0.91-1.09)	1.00	0.89 (0.82-0.97)	0.97 (0.90-1.05)	0.90 (0.82-0.98)	0.029	0.98 (0.97-1.00)
**ER+ and PR+ subtype**	2142	386	602	363	416	375		
Adjusted Hazard Ratio (95% CI)^f^		0.93 (0.80-1.08)	1.00	0.85 (0.74-0.98)	0.96 (0.84-1.10)	0.84 (0.73-0.97)	0.140	0.98 (0.96-0.99)
**ER- and PR- subtype**	605	126	154	116	104	105		
Adjusted Hazard Ratio (95% CI)^g^		1.14 (0.88-1.48)	1.00	0.89 (0.69-1.16)	0.81 (0.62-1.05)	0.80 (0.61-1.05)	0.008	0.96 (0.93-1.00)
**Analysis by cohort-wide intake**								
Adjusted Hazard Ratio (95% CI)^h^		1.12 (0.83-1.51)	1.00	1.17 (0.92-1.47)	0.97 (0.75-1.26)	1.11 (0.84-1.48)	0.989	1.00 (0.97-1.03)
Adjusted Hazard Ratio (95% CI)^i^		1.01 (0.92-1.12)	1.00	0.96 (0.88-1.04)	0.97 (0.89-1.06)	0.91 (0.83-1.00)	0.051	0.98 (0.97-1.00)

^a^Includes 226,368 participants with complete data on type of coffee intake, that is, France (n = 48,101), Germany (n = 27,411), Greece (n = 3,125), Italy (n = 11,737), Netherlands (n = 26,866), Norway (n = 35,170), Spain (n = 6,589), Sweden (n = 14,825), and United Kingdom (n = 52,544). ^b^Cut-off points are based on country specific quartiles of caffeinated coffee intake after exclusion of non-caffeinated coffee consumers. ^c^ *P* for trend is computed by entering the categories as a continuous term (score variable: 0,1,2,3,4) in the Cox model. ^d^Including only premenopausal breast cancers (that is, breast cancer diagnosed before the age of 50 years), and participants who were premenopausal at recruitment. Model is stratified by study center and age at recruitment, and adjusted for age at menarche, ever use of oral contraceptives, age at first delivery, breastfeeding, smoking, education, physical activity level, alcohol intake, height, weight, energy intake from fat sources, energy intake from non-fat sources, saturated fat intake, fruits and vegetable intake, decaffeinated coffee intake, and tea intake. ^e^Including only postmenopausal breast cancers (excluding participants with premenopausal breast cancers). Model is stratified by study center and age at recruitment, and adjusted for age at menarche, ever use of oral contraceptives, age at first delivery, breastfeeding, menopausal status at recruitment, ever use of postmenopausal hormones, smoking, education, physical activity level, alcohol intake, height, weight, energy intake from fat sources, energy intake from non-fat sources, saturated fat intake, fruits and vegetable intake, decaffeinated coffee intake, and tea intake. ^f^Hormone receptor status was only known in approximately 67% of patients with breast cancer. This analysis includes only estrogen receptor positive and progesterone receptor positive postmenopausal breast cancers, fully adjusted as in model 5. ^g^Hormone receptor status was only known in approximately 67% of patients with breast cancer. This analysis includes only estrogen receptor negative and progesterone receptor negative postmenopausal breast cancers, fully adjusted as in model 5. ^h^Including only premenopausal breast cancers. Using caffeinated coffee intake in cohort wide categories (no intake, quartile 1, quartile 2, quartile 3, quartile 4), and fully adjusted as in model 4. ^i^Including only postmenopausal breast cancers. Using caffeinated coffee intake in cohort wide categories (no intake, quartile 1, quartile 2, quartile 3, quartile 4), and fully adjusted as in model 5. CI, confidence interval, ER, estrogen receptor; PR, progesterone receptor.

**Table 4:** **Decaffeinated coffee consumption and risk of breast cancer** ^a^

**Daily decaffeinated coffee intake**	**Total**	**No intake of decaffeinated coffee**	**Low intake** ^b^	**Moderate intake** ^b^	**High intake** ^b^	***P***_trend_^c^	**Per 100 mls**
No. of participants	176373	88868	43173	16798	27534		
No. of breast cancers	5272	2858	1088	515	811		
**Premenopausal breast cancers**	587	289	149	48	101		
Adjusted Hazard Ratio (95% CI)^d^		1.08 (0.79-1.49)	1.00	0.86 (0.56-1.32)	1.20 (0.90-1.60)	0.646	1.00 (0.94-1.06)
**Postmenopausal cancers**	4685	2569	939	467	710		
Adjusted Hazard Ratio (95% CI)^e^		0.89 (0.80-0.99)	1.00	1.01 (0.89-1.15)	0.97 (0.87-1.08)	0.128	1.01 (0.99-1.03)
**ER+ and PR+ subtype**	1749	1073	280	174	222		
Adjusted Hazard Ratio (95% CI)^f^		0.88 (0.73-1.05)	1.00	0.98 (0.79-1.21)	1.07 (0.89-1.30)	0.036	1.02 (0.99-1.06)
**ER- and PR- subtype**	512	304	93	51	64		
Adjusted Hazard Ratio (95% CI)^g^		0.69 (0.50-0.94)	1.00	0.92 (0.63-1.34)	0.72 (0.51-1.02)	0.705	0.97 (0.91-1.04)
**Analysis by cohort-wide intake**							
Adjusted Hazard Ratio (95% CI)^h^		1.08 (0.76-1.53)	1.00	0.88 (0.62-1.27)	1.16 (0.84-1.60)	0.915	1.00 (0.94-1.06)
Adjusted Hazard Ratio (95% CI)^i^		0.87 (0.75-1.01)	1.00	1.00 (0.86-1.16)	0.95 (0.83-1.10)	0.081	1.01 (0.99-1.03)

^a^Includes 176,373 participants with complete data on type of coffee intake, that is, France (n = 48,101), Germany (n = 27,411), Greece (n = 3,125), Italy (n = 11,737), Netherlands (n = 26,866), Spain (n = 6,589), and United Kingdom (52,544). Participants from Norway and Sweden are all non-consumers of decaffeinated coffee and were excluded. ^b^Cut-off points are based on country specific tertiles of decaffeinated coffee intake after exclusion of non-decaffeinated coffee consumers. ^c^ *P* for trend is computed by entering the categories as a continuous term (score variable: 0,1,2,3,4) in the Cox model. ^d^Including only premenopausal breast cancers (that is, breast cancer diagnosed before the age of 50 years), and participants who were premenopausal at recruitment. Model is stratified by study center and age at recruitment, and adjusted for age at menarche, ever use of oral contraceptives, age at first delivery, breastfeeding, smoking, education, physical activity level, alcohol intake, height, weight, energy intake from fat sources, energy intake from non-fat sources, saturated fat intake, fruits and vegetable intake, caffeinated coffee intake, and tea intake. ^e^Including only postmenopausal breast cancers (excluding participants with premenopausal breast cancers). Model is stratified by study center and age at recruitment, and adjusted for age at menarche, ever use of oral contraceptives, age at first delivery, breastfeeding, menopausal status at recruitment, ever use of postmenopausal hormones, smoking, education, physical activity level, alcohol intake, height, weight, energy intake from fat sources, energy intake from non-fat sources, saturated fat intake, fruits and vegetable intake, caffeinated coffee intake, and tea intake. ^f^Hormone receptor status was only known in approximately 67% of patients with breast cancer. This analysis includes only estrogen receptor positive and progesterone receptor positive postmenopausal breast cancers, fully adjusted as in model 5. ^g^Hormone receptor status was only known in approximately 67% of patients with breast cancer. This analysis includes only estrogen receptor negative and progesterone receptor negative postmenopausal breast cancers, fully adjusted as in model 5. ^h^Including only premenopausal breast cancers. Using caffeinated coffee intake in cohort wide categories (no intake, quartile 1, quartile 2, quartile 3, quartile 4), and fully adjusted as in model 4. ^i^Including only postmenopausal breast cancers. Using caffeinated coffee intake in cohort wide categories (no intake, quartile 1, quartile 2, quartile 3, quartile 4), and fully adjusted as in model 5. CI, confidence interval, ER, estrogen receptor; PR, progesterone receptor.

**Table 5:** **Cross-classified coffee intake and risk of postmenopausal breast cancer** ^a^

		**Decaffeinated coffee**	
**Caffeinated coffee**		**No consumption**	**Consumption**
No consumption	Number of postmenopausal breast cancers/Number of participants	568/21239	287/9810
	Adjusted hazard ratio (95%CI)^c^	0.89 (0.77-1.04)	0.97 (0.82-1.14)
Low consumption^b^	Number of postmenopausal breast cancers/Number of participants	625/20480	601/29716
	Adjusted hazard ratio (95%CI)^c^	0.88 (0.77-1.02)	1.00
Moderate consumption^b^	Number of postmenopausal breast cancers/Number of participants	836/27498	588/22347
	Adjusted hazard ratio (95%CI)^c^	0.84 (0.74-0.97)	0.95 (0.83-1.08)
High consumption^b^	Number of postmenopausal breast cancers/Number of participants	540 19561	630/25632
	Adjusted hazard ratio (95%CI)^c^	0.82 (0.71-0.95)	0.98 (0.87-1.11)

^a^Includes 176,373 participants with complete data on type of coffee intake, that is, France (n = 48,101), Germany (n = 27,411), Greece (n = 3,125), Italy (n = 11,737), Netherlands (n = 26,866), Spain (n = 6,589), and United Kingdom (52,544). Participants from Norway and Sweden are all non- consumers of decaffeinated coffee and were excluded. ^b^The cut-off values are based on country specific tertiles. ^c^Includes only postmenopausal breast cancers. Model is stratified by study center and age at recruitment, and adjusted for age at menarche, ever use of oral contraceptives, age at first delivery, breastfeeding, menopausal status, ever use of postmenopausal hormones, smoking, education, physical activity level, alcohol intake, height, weight, energy intake from fat sources, energy intake from non-fat sources, saturated fat intake, fruits and vegetable intake, and tea intake. CI, confidence interval.

**Table 6:** **Tea consumption and risk of breast cancer** ^a^

**Daily tea intake**	**Total**	**No intake**	**Low intake** ^b^	**Moderately low intake** ^b^	**Moderately high intake** ^b^	**High intake** ^b^	***P***_trend_^c^	**Per 100 mls**
Number of participants	299890	99667	58966	54485	52280	34492		
Number of breast cancers	9344	3043	1704	1738	1680	1179		
**Premenopausal breast cancers**								
Adjusted Hazard Ratio (95% CI)^d^		0.90 (0.73-1.12)	1.00	0.98 (0.80-1.21)	0.97 (0.79-1.20)	0.98 (0.77-1.26)	0.624	1.00 (0.98-1.03)
**Postmenopausal cancers**	8407	2771	1486	1566	1510	1074		
Adjusted Hazard Ratio (95% CI)^e^		0.99 (0.92-1.06)	1.00	1.00 (0.93-1.08)	0.98 (0.91-1.06)	0.95 (0.88-1.03)	0.375	1.00 (0.99-1.00)
**ER+ and PR+ subtype**	2817	903	496	477	543	398		
Adjusted Hazard Ratio (95% CI)^f^		1.03 (0.91-1.15)	1.00	0.98 (0.86-1.11)	1.05 (0.93-1.19)	1.02 (0.89-1.17)	0.866	1.00 (0.99-1.02)
**ER- and PR- subtype**	959	268	177	182	180	152		
Adjusted Hazard Ratio (95% CI)^g^		1.12 (0.91-1.38)	1.00	0.99 (0.80-1.22)	1.03 (0.83-1.27)	1.12 (0.89-1.42)	0.941	1.00 (0.98-1.02)
**Analysis by cohort-wide intake**								
Adjusted Hazard Ratio (95% CI)^h^		0.91 (0.74-1.13)	1.00	1.04 (0.85-1.27)	0.94 (0.75-1.17)	0.97 (0.75-1.25)	0.770	1.00 (0.98-1.03)
Adjusted Hazard Ratio (95% CI)^i^		1.01 (0.93-1.09)	1.00	1.01 (0.94-1.10)	1.01 (0.93-1.10)	0.99 (0.91-1.08)	0.998	1.00 (0.99-1.00)

^a^Includes 299890 participants, following exclusion of participants from Norway where data on tea intake is not available. ^b^Cut-off points are based on country specific quartiles of tea intake after exclusion of non-tea consumers. ^c^ *P* for trend is computed by entering the categories as a continuous term (score variable: 0,1,2,3,4) in the Cox model. ^d^Including only premenopausal breast cancers (that is, breast cancer diagnosed before the age of 50 years), and participants who were premenopausal at recruitment. Model is stratified by study center and age at recruitment, and adjusted for age at menarche, ever use of oral contraceptives, age at first delivery, breastfeeding, smoking, education, physical activity level, alcohol intake, height, weight, energy intake from fat sources, energy intake from non-fat sources, saturated fat intake, fruits and vegetable intake, coffee intake. ^e^Including only postmenopausal breast cancers (excluding participants with premenopausal breast cancers). Model is stratified by study center and age at recruitment, and adjusted for age at menarche, ever use of oral contraceptives, age at first delivery, breastfeeding, menopausal status at recruitment, ever use of postmenopausal hormones, smoking, education, physical activity level, alcohol intake, height, weight, energy intake from fat sources, energy intake from non-fat sources, saturated fat intake, fruits and vegetable intake, coffee intake. ^f^Hormone receptor status was only known in approximately 67% of patients with breast cancer. This analysis includes only estrogen receptor positive and progesterone receptor positive postmenopausal breast cancers, fully adjusted as in model 5. ^g^Hormone receptor status was only known in approximately 67% of patients with breast cancer. This analysis includes only estrogen receptor negative and progesterone receptor negative postmenopausal breast cancers, fully adjusted as in model 5. ^h^Including only premenopausal breast cancers. Using tea intake in cohort wide categories (no intake, quartile 1, quartile 2, quartile 3, quartile 4), and fully adjusted as in model 4. ^i^Including only postmenopausal breast cancers. Using tea intake in cohort wide categories (no intake, quartile 1, quartile 2, quartile 3, quartile 4), and fully adjusted as in model 5. CI, confidence interval, ER, estrogen receptor; PR, progesterone receptor.

### Total coffee

While moderately low intake of total coffee consumption seemed to be associated with higher risk of premenopausal breast cancer (adjusted HR:1.23, 95% CI: 1.02 to 1.48, compared to low intake), no dose response relationship was observed; *P*_trend_ = 0.272 (Table 2). Overall, intake of total coffee was associated with a borderline statistically significantly lower risk of postmenopausal breast cancer. Multivariable HR comparing high total coffee intake to low intake was 0.95 (95% CI: 0.89 to 1.01). The linear trend test was not significant; *P*_trend_ = 0.055. Each 100 ml increase in daily intake of total coffee was inversely associated with breast cancer risk (HR continuous 0.99, 95% CI: 0.98 to 0.99).

### Caffeinated coffee

There seemed to be no association between caffeinated coffee intake and premenopausal breast cancer (Table 3). However, higher intakes of caffeinated coffee were associated with lower risk of postmenopausal breast cancer (adjusted HR for high intake compared to low intake: 0.90; 95% CI: 0.82 to 0.98). A linear trend for the inverse associations of caffeinated coffee intake with postmenopausal breast cancer risk was also apparent in this analysis; *P*_trend_ = 0.029 (Table 3). While there was no significant effect modification by hormone receptor status (*P* = 0.711), a linear trend for lower risk of breast cancer with increasing caffeinated coffee intake was clearest for ER- PR- breast cancer (*P* = 0.008). For every 100 ml higher caffeinated coffee consumption, the risk of ER- PR- breast cancer was lower by 4% (adjusted HR: 0.96, 95% CI: 0.93 to 1.00). The risk of ER+ PR+ breast cancer was lowered by 2% per 100 ml (adjusted HR: 0.98, 95% CI: 0.96 to 0.99). However, the *P* value for trend test of categorical analyses was not significant (Table 3).

### Decaffeinated coffee

No association was observed between decaffeinated coffee intake and premenopausal breast cancer (Table 4). Non-consumers compared to low consumers of decaffeinated coffee did show a significantly lower postmenopausal breast cancer risk (adjusted HR: 0.89; 95% CI: 0.80 to 0.99). There was, however, no difference in risk of breast cancer between high decaffeinated coffee consumers, compared to low consumers (Table 4). *Post-hoc* analysis comparing non-consumers of decaffeinated coffee against the consumers (no intake versus any intake, irrespective of caffeinated coffee intake) showed a modest decrease in risk of postmenopausal breast cancer; adjusted HR: 0.90, 95% CI: 0.82 to 0.98. There was no dose response relationship (*P*_trend_ = 0.128).

Compared to low decaffeinated coffee consumers, it seemed that the non-consumers of decaffeinated coffee had a lower risk of developing ER- PR- breast cancer (adjusted HR:0.69, 95% CI: 0.50 to 0.94), than ER+ PR+ breast cancer (adjusted HR: 0.88, 95% CI:0.73 to 1.05). However, test for interaction with hormone receptor status was not statistically significant (*P* = 0.716).

### Cross-classification of caffeinated and decaffeinated coffee

As compared to decaffeinated coffee consumers with low caffeinated coffee intake, non-consumers of decaffeinated coffee with higher intakes of caffeinated coffee had significantly lower risk of postmenopausal breast cancer. Within consumers of decaffeinated coffee, no such association with higher intakes of caffeinated coffee was observed. Exclusive decaffeinated coffee consumption was also not associated with risk of postmenopausal breast cancer compared to decaffeinated coffee consumption with low caffeinated coffee intake (Table 5). *Post-hoc* analysis within women who did not consume any caffeinated coffee, showed no difference in risk of postmenopausal breast cancer between 21,239 non-consumers of decaffeinated coffee and 9,810 decaffeinated coffee consumers (adjusted HR: 0.96; 95%: 0.82 to 1.14).

### Tea

Tea consumption was neither statistically significantly associated with risk of premenopausal nor postmenopausal breast cancer (Table 6). The adjusted HR for high tea intake versus low intake was 0.98 (95% CI: 0.77 to 1.26) for premenopausal breast cancer, and 0.95 (95% CI: 0.88 to 1.03) for postmenopausal breast cancer. Analysis by hormone receptor status did not show any significant results.

### Sensitivity analysis

Our sensitivity analyses showed that results remain essentially unchanged when analysis of beverage intake was conducted using cohort wide categories of intake instead of country specific categories (Tables 2, 3, 4 and 6). We did not observe any effect modification by BMI. There was no statistically significant heterogeneity between countries for the association between total coffee, caffeinated coffee, decaffeinated coffee, and tea intake and breast cancer. None of the associations were substantially altered when family history of breast cancer was included in the analyses, or when analysis was restricted to follow-up experience after two years of recruitment into the study (not shown).

## Discussion

In this study, high versus low caffeinated coffee intake was associated with a modest but statistically significantly lower risk of postmenopausal breast cancer. This association was only detected in women not consuming decaffeinated coffee. Although abstinence of decaffeinated coffee (versus any intake, irrespective of caffeinated coffee intake) seemed to be associated with lower risk for postmenopausal breast cancer, exclusive decaffeinated coffee intake was not associated with increased risk. Tea intake was not associated with risk of postmenopausal breast cancer. Neither caffeinated coffee, decaffeinated coffee, nor tea intake impacted the risk of premenopausal breast cancer.

Our null finding for the association between total coffee intake and risk of postmenopausal breast cancer corroborates the findings of most previous large-scale prospective studies and meta-analyses [12,28]. The lack of association observed in the current study and previous studies seems to support the notion that studying total coffee intake as a single entity may result in a net null association owing to differences in the direction of association between caffeinated and decaffeinated coffee, in relation to breast cancer. Hence, we would like to recommend that future studies in populations consuming both types of coffee should explicitly analyze caffeinated and decaffeinated coffee intake separately.

The observation that caffeinated coffee was significantly associated with lower risk of postmenopausal breast cancer seems to be in agreement with the finding of a recent population based case–control study by Lowcock *et al*. in Canada (odds ratio comparing highest versus no consumption: 0.63 (95% CI: 0.43 to 0.94) [19]. This study, and another population-based case–control study, which included participants from Sweden as well as Germany [29], had further found that caffeinated coffee intake was significantly associated with a reduced risk of estrogen receptor negative breast cancers but not estrogen receptor positive breast cancers, while we found a stronger association in ER- PR- breast cancers. A cohort study in Sweden had also found that increased coffee intake was associated with lower risk of ER- PR- breast cancer, but at a more modest margin of protection, not achieving statistical significance [30]. Other recent prospective cohort studies, however, could not show an association between caffeinated coffee intake and risk of breast cancer [31-35]. While it seems plausible that caffeine plays a role in explaining the lower risk of breast cancer associated with caffeinated coffee intake in the current study [35], a number of studies have shown that caffeine intake *per se* does not impact breast cancer risk [19,32-34,36]. It is hence postulated that another compound, or compounds, in caffeinated coffee may confer a protection against breast carcinogenesis by acting synergistically with caffeine [19]. This explanation seems plausible given that in our study, caffeinated coffee, which contains caffeine and a plethora of other compounds, was associated with lower risk of postmenopausal breast cancer, whereas decaffeinated coffee does not seem to be associated with risk of breast cancer.

In this study, those who reported not to consume decaffeinated coffee seemed to have a significantly lower risk of breast cancer. However, we did not observe a dose–response relationship. Cross-classified coffee intake analysis further showed that exclusive decaffeinated coffee consumption was not associated with risk of postmenopausal breast cancer compared to decaffeinated coffee and low caffeinated coffee consumption. *Post-hoc* analyses also showed that exclusive decaffeinated coffee consumption was not associated with increased risk of postmenopausal breast cancer compared to no intake of any coffee. Taken together, these findings seem to suggest that decaffeinated coffee intake is not associated with postmenopausal breast cancer. The apparent decrease in risk among non-consumers of decaffeinated coffee may be explained by findings of previous studies, which have suggested that decaffeinated coffee consumers may be unique in terms of lifestyle or medical history [37,38]. Decaffeinated coffee intake is related to illness in some persons but to a healthy lifestyle in others [37]. This is corroborated in the current study, where consumption of decaffeinated coffee was associated with a healthier lifestyle compared to non-consumption. Hence, there may have been minimal overlap in (lifestyle related) confounders between the consumers and non-consumers of decaffeinated coffee to allow optimal adjustment. It is also conceivable that the breast cancer screening behavior of decaffeinated coffee consumers may have contributed to higher detection of (early) breast cancers in this subgroup. This may also explain why higher caffeinated coffee intakes within decaffeinated coffee consumers were not associated with a lower risk of breast cancer. It is, however, acknowledged that distinguishing genuine decaffeinated coffee effects from a ‘decaffeinated coffee preference’ effect may be difficult.

A meta-analysis on the association of tea intake with breast cancer risk had found no overall protective effect of black tea (pooled relative risk = 0.97; 95% CI = 0.91 to 1.05) [3]. This corroborates our findings since black tea is the predominantly consumed type of tea in Europe [39]. Possibly explaining the lack of association between black tea and breast cancer is that it contains a relatively lower amount of caffeine compared to coffee, and up to 10-fold reduction in catechin levels compared with green tea, which had been inversely associated with breast cancer [39].

Besides having a sufficiently large number of premenopausal breast cancers, we also had a relatively high number of breast cancer cases whose hormone receptor statuses were available (approximately 70%) to allow analysis by ER and PR status. Our findings further support the view that pooling of pre- and postmenopausal breast cancers as a homogenous entity is not recommended. We do, however, acknowledge that data on menopausal status at diagnosis was not available, and we had to use age at breast cancer diagnosis as a proxy. Although coffee and tea intakes were measured only at baseline, analyses of participants in the EPIC sub-cohort of Umea in Sweden [40], as well as participants of the Cancer Prevention Study II in the United States [41], with repeated measures taken up to 10 years apart showed that coffee habits are stable over a long period. While the amount of coffee intake seems to vary substantially across Europe, true variation in coffee intake may not be as large as it seems given that there is an inverse relationship between the volume and concentration of coffee. This is reflected in our results whereby the hazard ratios using cohort-wide cutpoints are similar to country-specific cutpoints. We did not have information on the type of tea, and coffee/tea brewing methods, which may vary across the countries and alter the contents of potentially beneficial compounds in the beverages. It has been recently highlighted that coffee brewing methods maybe be relevant with respect to breast cancer risk. A cohort study in Sweden showed that a decreased risk of breast cancer was observed in women drinking boiled coffee but no association was observed with filtered coffee consumption [42]. Country specific categories of consumption were therefore used to address this limitation. Besides coffee and tea as prominent sources of caffeine in this population, hot chocolate, chocolate candy/candy bars, and soft drinks are also possible sources [43]. We had information on chocolate candy/candy bars intake and soft drinks, but not on hot chocolate intake. As contributions of caffeine from these sources are far lower than from coffee and tea, those intakes are unlikely to have impacted our study results [43].

## Conclusions

Within a very large cohort of women, our findings show that higher caffeinated coffee intake is associated with a modest lowering in risk of postmenopausal breast cancer. Decaffeinated coffee intake does not seem to be associated with risk of breast cancer. The mechanism by which caffeinated coffee impacts breast cancer risk warrants further investigation.

## Additional file

Additional file 1:
**Supplementary methods.** Cox regression models for pre- and postmenopausal breast cancers.

## Abbreviations

BMI: body mass index
CI: confidence interval
EPIC: European Prospective Investigation into Nutrition and Cancer
ER: estrogen receptor
HR: hazard ratio
IARC: International Agency for Research on Cancer
PR: progesterone receptor

## References

[1] Holick CN, Smith SG, Giovannucci E, Michaud DS (2010). Coffee, tea, caffeine intake and risk of adult glioma in 3 prospective cohort studies. Cancer Epidemiol Biomarkers Prev..

[2] Nkondjock A (2009). Coffee consumption and the risk of cancer: an overview. Cancer Lett..

[3] Yang CS, Wang X, Lu G, Picinich SC (2009). Cancer prevention by tea: animal studies, molecular mechanisms and human relevance. Nat Rev Cancer..

[4] Cavin C, Holzhaeuser D, Scharf G, Constable A, Huber WW, Schilter B (2002). Cafestol and kahweol, two coffee specific diterpenes with anticarcinogenic activity. Food Chem Toxicol..

[5] Kotsopoulos J, Eliassen AH, Missmer SA, Hankinson SE, Tworoger SS (2009). Relationship between caffeine intake and plasma sex hormone concentrations in premenopausal and postmenopausal women. Cancer..

[6] Jernström H, Klug TL, Sepkovic DW, Bradlow HL, Narod SA (2003). Predictors of the plasma ratio of 2-hydroxyestrone to 16alpha-hydroxyestrone among pre-menopausal, nulliparous women from four ethnic groups. Carcinogenesis..

[7] Woolcott CG, Shvetsov YB, Stanczyk FZ, Wilkens LR, White KK, Caberto C (2010). Plasma sex hormone concentrations and breast cancer risk in an ethnically diverse population of postmenopausal women: the Multiethnic Cohort Study. Endocr Relat Cancer..

[8] Kaaks R, Rinaldi S, Key TJ, Berrino F, Peeters PH, Biessy C (2005). Postmenopausal serum androgens, oestrogens and breast cancer risk: the European prospective investigation into cancer and nutrition. Endocr Relat Cancer..

[9] Walker K, Bratton DJ, Frost C (2011). Premenopausal endogenous oestrogen levels and breast cancer risk: a meta-analysis. Br J Cancer..

[10] Rice S, Whitehead SA (2006). Phytoestrogens and breast cancer –promoters or protectors?. Endocr Relat Cancer..

[11] World Cancer Research Fund (2007). Food, nutrition, physical activity, and the prevention of cancer: a global perspective.

[12] Jiang W, Yu Y, Jiang X (2013). Coffee and caffeine intake and breast cancer risk: an updated dose–response meta-analysis of 37 published studies. Gynecol Oncol..

[13] Decaffeination. International Coffee Organisation, London. http://www.ico.org/Decaffeination.asp. Accessed 9 Sep 2014.

[14] Clavel-Chapelon F, EBM-EPIC Group (2002). Differential effects of reproductive factors on the risk of pre- and postmenopausal breast cancer. Results from a large cohort of French women. Br J Cancer..

[15] Lubin JH, Burns PE, Blot WJ, Lees AW, May C, Morris LE (1982). Risk factors for breast cancer in women in northern Alberta, Canada, as related to age at diagnosis. J Natl Cancer Inst..

[16] Cho E, Chen WY, Hunter DJ, Stampfer MF, Colditz GA, Hankinson SE (2006). Red meat intake and risk of breast cancer among premenopausal women. Arch Intern Med..

[17] Anderson WF, Rosenberg PS, Prat A, Perou CM, Sherman ME. How many etiological subtypes of breast cancer: two, three, four, or more? J Natl Cancer Inst. 2014;106. doi: 10.1093/jnci/dju165. 10.1093/jnci/dju165 PMC4148600 25118203

[18] Yu Y, Zhang D, Kang S (2013). Black tea, green tea and risk of breast cancer: an update. Springerplus..

[19] Lowcock EC, Cotterchio M, Anderson LN, Boucher BA, El-Sohemy A (2013). High coffee intake, but not caffeine, is associated with estrogen receptor negative and postmenopausal breast cancer risk with no effect modification by CYP1A2 genotype. Nutr Cancer..

[20] Bhoo-Pathy N, Peeters P, van Gils C, Beulens JW, van der Graaf Y, Bueno-de-Mesquita B (2010). Coffee and tea intake and risk of breast cancer. Breast Cancer Res Treat..

[21] Riboli E, Hunt KJ, Slimani N, Ferrari P, Norat T, Fahey M (2002). European Prospective Investigation into Cancer and Nutrition (EPIC): study populations and data collection. Public Health Nutr..

[22] Margetts BM, Pietinen P (1997). European Prospective Investigation into Cancer and Nutrition: validity studies on dietary assessment methods. Int J Epidemiol..

[23] Wareham NJ, Jakes RW, Rennie KL, Schuit J, Mitchell J, Hennings S (2003). Validity and repeatability of a simple index derived from the short physical activity questionnaire used in the European Prospective Investigation into Cancer and Nutrition (EPIC) study. Public Health Nutr..

[24] Slimani N, Kaaks R, Ferrari P, Casagrande C, Clavel-Chapelon F, Lotze G (2002). European Prospective Investigation into Cancer and Nutrition (EPIC) calibration study: rationale, design and population characteristics. Public Health Nutr..

[25] Ferrari P, Day NE, Boshuizen HC, Roddam A, Hoffmann K, Thiébaut A (2008). The evaluation of the diet/disease relation in the EPIC study: considerations for the calibration and the disease models. Int J Epidemiol..

[26] Lunn M, McNeil D (1995). Applying Cox regression to competing risks. Biometrics..

[27] Vatten LJ, Solvoll K, Loken EB (1990). Coffee consumption and the risk of breast cancer. A prospective study of 14, 593 Norwegian women. Br J Cancer.

[28] Tang N, Zhou B, Wang B, Yu R (2009). Coffee consumption and risk of breast cancer: a meta-analysis. Am J Obstet Gynecol.

[29] Li J, Seibold P, Chang-Claude J, Flesch-Janys D, Liu J, Czene K (2011). Coffee consumption modifies risk of estrogen receptor negative breast cancer. Breast Cancer Res..

[30] Larsson SC, Bergkvist L, Wolk A (2009). Coffee and black tea consumption and risk of breast cancer by estrogen and progesterone receptor status in a Swedish cohort. Cancer Causes Control..

[31] Gierarch GL, Freedman ND, Andaya A, Hollenbeck AR, Park Y, Schatzkin A (2012). Coffee intake and breast cancer risk in the NIH-AARP diet and health study cohort. Int J Cancer..

[32] Fagherazzi G, Touillaud MS, Boutron-Ruault MC, Clavel-Chapelon F, Romieu I (2011). No association between coffee, tea, or caffeine consumption and breast cancer risk in a prospective cohort study. Public Health Nutr..

[33] Boggs DA, Palmer JR, Stampfer MJ, Spiegelman D, Adams-Campbell LL, Rosenberg L (2010). Tea and coffee intake in relation to risk of breast cancer in the Black Women’s Health Study. Cancer Causes Control..

[34] Ishitani K, Lin J, Manson JE, Buring JE, Zhang SM (2008). Caffeine consumption and the risk of breast cancer in a large prospective cohort of women. Arch Intern Med..

[35] Ganmaa D, Willet WC, Li TY, Feskanich D, van Dam RM, Lopez-Garcia E (2008). Coffee, tea, caffeine and risk of breast cancer: a 22-year follow-up. Int J Cancer..

[36] Michels KB, Holmberg L, Bergkvist L, Wolk A (2002). Coffee, tea, and caffeine consumption and breast cancer incidence in a cohort of Swedish Women. Ann Epidemiol..

[37] Shlonsky AK, Klatsky AL, Armstrong MA (2003). Traits of persons who drink decaffeinated coffee. Annals Epidemiol..

[38] Leviton A, Allred EN (1994). Correlates of decaffeinated coffee. Epidemiology..

[39] Sun CL, Yuan JM, Koh WP, Yu MC (2006). Green tea, black tea and breast cancer risk: a meta-analysis of epidemiological studies. Carcinogenesis..

[40] Nilsson LM, Wennberg M, Lindahl B, Elliason M, Jansson JH, Van Guelpen B (2009). Consumption of filtered and boiled coffee and the risk of first acute myocardial infarction; a nested case study. Nutr Metab Cardiovasc Dis..

[41] Hildebrand JS, Patel AV, McCullough ML, Gaudet MM, Chen AY, Hayes RB (2013). Coffee, tea, and fatal oral/ pharyngeal cancer in a large prospective US cohort. Am J Epidemiol..

[42] Nilsson LM, Johansson I, Lenner P, Lindahl B, Van Guelpen B (2010). Consumption of filtered and boiled coffee and the risk of incident cancer: a prospective cohort study. Cancer Causes Control..

[43] Heckman MA, Weil J (2010). Gonzalez de Mejia E. Caffeine (1, 3, 7-trimethylxanthine) in foods: a comprehensive review on consumption, functionality, safety, and regulatory matters. J Food Sci..

