# Supplementary material for: Coffee and tea consumption and risk of pre- and postmenopausal breast cancer in the European Prospective Investigation into Cancer and Nutrition (EPIC) cohort study
Source: Breast Cancer Res. 2015 Jan 31;17(1):15. doi: 10.1186/s13058-015-0521-3 (PMC4349221; doi:10.1186/s13058-015-0521-3)
Supplement: Additional file 1: — Supplementary methods. Cox regression models for pre- and postmenopausal breast cancers. [file 13058_2015_521_MOESM1_ESM.docx]

**SUPPLEMENTARY METHODS**

**Cox regression models for pre- and postmenopausal breast cancers:**

Premenopausal breast cancers

In the premenopausal model, women aged ≥ 50 years at recruitment were excluded, as well as those reporting to have attained menopause at baseline. Exit time was age at diagnosis of breast cancer as the first tumor (before attaining 50 years of age), or turning 50 years, death, emigration, loss to follow-up, or end of follow-up, whichever came first. Models were adjusted for age at menarche, ever use of oral contraceptives, age at first delivery, ever breastfeeding, smoking status, educational level, physical activity level based on Cambridge Physical Activity Index^16^, height, weight, energy intake from fat source, energy intake from non-fat source, total saturated fat intake, and total fiber intake. Coffee and tea intake were mutually adjusted for one another while models for caffeinated and decaffeinated coffee were also mutually adjusted

Postmenopausal breast cancers

Analyses of postmenopausal breast cancers, excluded premenopausal breast cancers (n=1064), leaving 333,996 women. Models were adjusted similar to the premenopausal model, as well as for ever-use of postmenopausal hormones (yes/no). Subgroup analysis for postmenopausal breast cancer was performed by hormone receptor status: ER+PR+, ER+PR-, ER-PR- breast cancers.

**Calibration of coffee and tea intake:**

Calibration

Dietary intake was calibrated by a 24 -hour dietary recall method common to all centers. Face-to-face 24-hour recall interviews were done using a computerized program (EPIC-SOFT), to adjust for systematic and random intra-individual error and between-center errors. Linear regression calibration^18^ was done with 24-hour dietary recall measurements being regressed on dietary questionnaire values for coffee (total, caffeinated, decaffeinated) and tea consumption, to obtain predicted intake values.

In the calibration models, the same covariates as in the Cox regression models described above were included. Additionally, data were weighted by day of the week and season of the year on which the 24-hour dietary recall was obtained. Cox regression models were then fitted with continuous predicted values (100ml/day) to obtain the measurement error-corrected (de-attenuated) HR estimates. To account for additional variability introduced by the calibration model, the standard errors of the de-attenuated coefficients were corrected through bootstrap sampling (10 repetitions).

**Additional analyses:**

Effect modification

Since body mass index (BMI) might modify the association between coffee intake and risk of breast cancer, likelihood ratio tests were applied between nested models with and without multiplicative interaction terms using continuous values to assess for possible effect modification.

Heterogeneity

To assess for heterogeneity of estimates across countries, we introduced an interaction term; ‘countries multiplied by beverage intakes (continuous)’ into the model.

In order to assess heterogeneity of the association by hormone receptor status, we used the data-augmentation method. In this analysis, we included only breast cancer cases of which information on hormone receptor status was available. We compared the difference in the log likelihood between a model with receptor status–specific variable and a model with a single hazard ratio estimate for the 2 categories of receptor status together with chi-square statistics with one degree of freedom (*p*_heterogeneity_).

Sensitivity analysis

In the 43% of women with available information on family history of breast cancer, analyses with and without adjustment for family history of breast cancer were performed. To preclude reverse causation by preclinical disease influencing coffee and tea consumption, we repeated the analysis excluding the first two years of follow-up.
